# ﻿Redescription of *Periplanetaarabica* (Bey-Bienko, 1938) (Blattodea, Blattidae), with a comparative analysis of three species of *Periplaneta* Burmeister, 1838 (*sensu stricto*)

**DOI:** 10.3897/zookeys.1146.90817

**Published:** 2023-02-09

**Authors:** Xin-Xing Luo, Qian-Qian Li, Alireza Zamani, Yan-Li Che, Zong-Qing Wang

**Affiliations:** 1 Institute of Entomology, College of Plant Protection, Southwest University, Beibei, Chongqing 400716, China Southwest University Chongqing China; 2 Zoological Museum, Biodiversity Unit, FI-20014 University of Turku, Turku, Finland University of Turku Turku Finland

**Keywords:** Blattid cockroach, DNA barcoding, female genitalia, habitat adaptation, male genitalia, sexual dimorphism, *Shelfordella*, taxonomy

## Abstract

The blattid cockroach *Periplanetaarabica* (Bey-Bienko, 1938) has been poorly understood since its original description. In this study, male and female (including nymph) of *P.arabica* are paired using DNA barcoding, and their morphological characters (including both external characteristics and genitalia) are described. A detailed comparative morphological study of this species and the closely related *Periplanetaamericana* (Linnaeus, 1758) and *Periplanetalateralis* Walker, 1868 was carried out to explore phylogenetically relevant characters.

## ﻿Introduction

According to Beccaloni (2014), the Blattinae genus *Shelfordella* Adelung, 1910 comprised three species before it was synonymized with *Periplaneta* (Deng et al. 2023). The taxonomic status of *Shelfordella* remains unclear even though several revisions were carried out by Princis (1954) and Bohn (1985) based on the external morphological characters. In addition, many molecular phylogenetic studies (Legendre et al. 2015; Bourguignon et al. 2018; Arab et al. 2020; Liao et al. 2021; Djernæs and Murienne 2022; Li et al. 2022; Deng et al. 2023) have shown that *Periplanetaamericana* (Linnaeus, 1758), the type species of *Periplaneta* Burmeister, 1838, is the sister species to *Sh.lateralis* (Walker, 1868). Considering both molecular data and morphological characters of male genitalia of *P.americana* and *Sh.lateralis*, *Shelfordella* was considered as a synonym of *Periplaneta* (Deng et al. 2023), resulting in the restoration of *Periplanetalateralis* Walker, 1868 and *Periplanetamonochroma* Walker, 1871, and the transference of *Shelfordellaarabica* Bey-Bienko, 1938 to *Periplaneta*. *Periplanetaarabica* was originally described with a female specimen as its type, and the male has not been described.

DNA barcoding has been confirmed to be a helpful tool in discovery of new species, matching nymphs with adults, and revealing sexual dimorphism and cryptic species in cockroaches (Evangelista et al. 2013; Che et al. 2017; Yang et al. 2019; Li et al. 2022; Zhu et al. 2022). Herein, we use DNA barcoding to pair male, female and nymphs of *P.arabica*, allowing a comprehensive redescription of this species. We also take the opportunity to compare the morphological characters of *P.arabica*, *P.americana* and *P.lateralis*, to show the structural complexity and diversity of species of *Periplanetas.s.*, as well as to provide detailed information useful for future phylogenetic studies on the genus.

## ﻿Material and methods

### ﻿Morphological study

Specimens (stored in absolute ethanol at -20 °C) examined are deposited in the Institute of Entomology, College of Plant Protection, Southwest University, Chongqing, China (SWU). Abdominal segments were soaked in 10% NaOH solution at 70 °C for 10 minutes. They were cleaned in distilled water, dissected in glycerol under a Motic K400 stereomicroscope, then stored in glycerol. Photographs were taken using a Canon M5 plus a Laowa 65 mm F2.8 CA-Dreamer Macro 2X Macro lens attached to a Leica M205A stereomicroscope. All figures were modified in Adobe Photoshop CC 2019. The morphological terminology used in this paper mainly follows Roth (2003). The terminology of veins follows Li et al. (2018), and those of the sclerites of male and female genitalia mainly follows Klass (1997) and McKittrick (1964), respectively. Measurements were obtained by Vernier Caliper.

### ﻿Abbreviations used are as follows:

**Cu** cubitus

**CuA** cubitus anterior

**CuP** cubitus posterior

**hlap** process (p) of hook of L3

**M** media

**Pcu** postcubitus

**R** radius

**RA** radius anterior

**RP** radius posterior

**ScP** subcostal posterior

**V, V[1], V[s]** vannal veins

**L1, L2, L3, L4C, L4D, L4EL4G** sclerites of the left phallomere

**R1G, R1H, R1F, R2, R3** sclerites of the right phallomere

### ﻿DNA extraction, amplification and sequencing

Total DNA extraction was obtained from muscle tissue using the Hipure Tissue DNA Mini Kit, and the remaining specimens were stored in 95% ethanol. The primers used to amplify the 658 bp cytochrome c oxidase subunit I (COI) fragment were COI-F2 (5’- CAACAAATCATAAAGATATTGGAAC-3’) and COI-R2 (5’- TAAACTTCTGGATGACCAAAAAATCA -3’) or COI-F3 (5’- CAACYAATCATAAAGANATTGGAAC -3’) and COI-R3 (5’-TAAACTTCAGGGTGACCAAARAATCA-3’) (Yang et al. 2019). The amplification reaction was in according to the protocols in Wang et al. (2021). The cycling conditions were as follows: initial denaturation at 98 °C for 2 min, followed by 35 cycles of 98 °C for 10 s, 49–51 °C for 10 s, 72 °C for 10 s, and a final extension at 72 °C for 10 min. The PCR products were then sequenced by BGI Technology Solutions Co. Ltd (BGI-Tech) (Beijing, China).

### ﻿Sequence processing and molecular analysis

A total of 25 COI sequences were analyzed, of which, 17 sequences were from three *Periplaneta* species (i.e., six sequences of *P.arabica*, five sequences of *P.americana* and six sequences of *P.lateralis*) (Table 1). Sequences were aligned by MAFFT ver. 7 (https://mafft.cbrc.jp/alignment/server/) with the G-INS-i strategy (Katoh et al. 2019), and manually adjusted using MEGA ver. 7.0.26 (Kumar et al. 2007). The intra- and interspecific genetic distances were quantified in MEGA based on the Kimura 2-parameter (K2P) distance model (Kimura 1980) (Suppl. materials 1, 2). The maximum likelihood (ML) tree was constructed in IQ-TREE ver. 1.6.8 (Nguyen et al. 2015) with 10,000 ultrafast bootstrap replicates; the partition scheme and best-fitting substitution models (COI_pos 1, TRN+I+G; COI_pos 2, TVM+I; COI_pos 3, HKY+I+G) were selected in PartionFinder ver. 2.1.1 (Lanfear et al. 2017) by the corrected Akaike Information Criterion (AICc).

**Table 1.** Samples used in ML analyses with localities, voucher numbers, and accession numbers (bold represent the new sequences). Abbreviations: young nymph (YN); late nymph (LN).

Species	Voucher number	Locality/References	Accession Number
*Periplanetaarabica*	1213(YN), 1208(♀), YL1(♂), SYL (♂), Shelarab1211(LN), YL2(♀)	Dehloran, Ilam, Iran	**OP727639** to **OP727640** and **OP727649** to **OP727652**
*Periplanetaamericana*		Bahamas: Exuma, Staniel (Pringle et al. 2019)	MK936745
	1416(♂),	Yuanjiang, Yunnan, China	**OP727642**
	1124(♂), 1417(♀)	Mt Diaoluo, Hainan, China	**OP727638** and **OP727643**
	1415(♀)	Meizhou Island, Fujian, China	**OP727641**
*Periplanetalateralis*	2401(♂), 2430(♀), 2433(♀), 2435(♀), 2440(♀)	Laboratory Rearing (online shopping)	**OP727644** and **OP727648**
		Breeds of Kyle Kandilian (Bourguignon et al. 2018)	MG882183
*Blattaorientalis*	–	Bourguignon et al. (2018)	MG882174
*Periplanetabrunnea*	–	Bourguignon et al. (2018)	MG882182
*Periplanetafuliginosa*	–	Ma et al. (2019)	MF149696
*Periplanetaaustralasiae*	–	Ma et al. (2019)	MH184379
*Cryptocercusmeridianus*	–	Li et al. (2017)	MG518617
*Tryonicusmackerrasae*	–	Bourguignon et al. (2018)	MG882205
*Hebardinaconcinna*	–	Deng et al. (2023)	ON645482
*Mantisreligiosa*	–	Ye et al. (2016)	NC030265

## ﻿Results

### ﻿Molecular analysis

In this study, we used six COI sequences of *P.arabica*, five COI sequences of *P.americana* and six COI sequences of *P.lateralis*. All new sequences were deposited in GenBank with accession numbers OP727638 to OP727652. Intraspecific COI genetic divergence (K2P) of *P.arabica* and *P.lateralis* is 0%, but for *P.americana*, the intraspecific COI genetic divergence ranged from 0.00% to 2.30%. Interspecific COI genetic divergence ranged from 9.9% (*P.arabica* and *P.americana*) to 13.1% (*P.americana* and *P.lateralis*).

**Figure 1.**
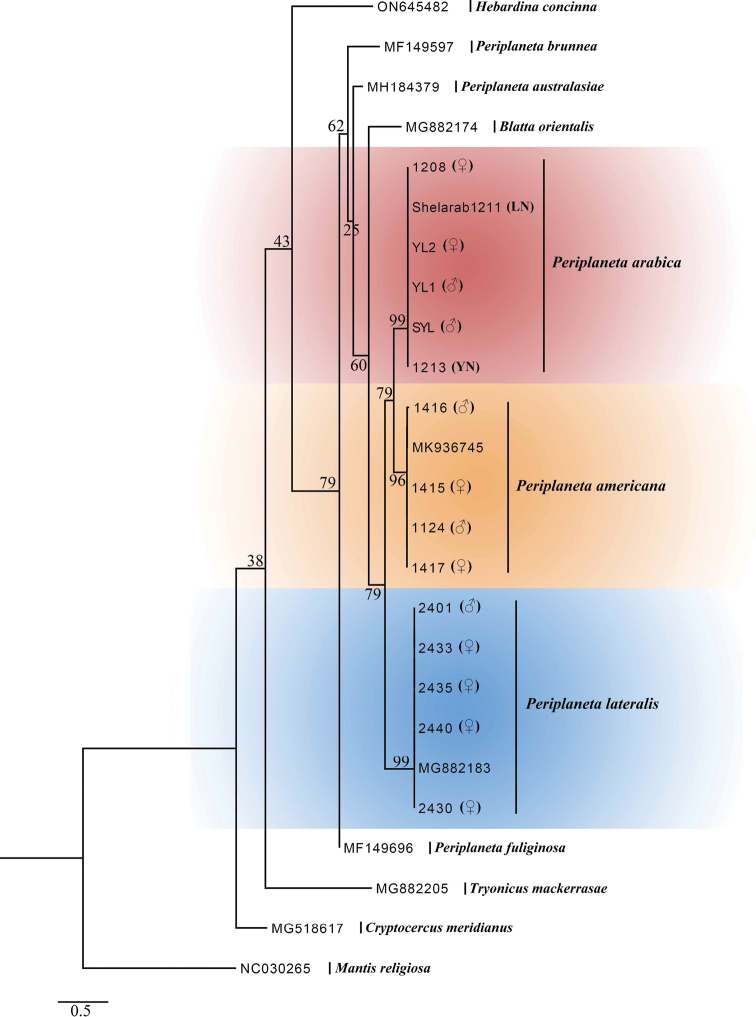
Maximum likelihood (ML) tree derived from COI sequences with 10,000 ultrafast bootstrap replicates.

In our ML analyses, samples including adults and nymphs from the same morphospecies are clustered together with high support values (Fig. 1). *Periplanetaarabica* was recovered as the sister to *P.americana* on the basis of COI data but with a rather low support (bootstrap support (BS) = 79). These three species (i.e., *P.arabica*, *P.lateralis* and *P.americana*) formed a monophyletic group with *Blattaorientalis* as the sister (BS = 79 and 60, respectively).

### ﻿Taxonomy

#### ﻿Genus Periplaneta

Taxon classificationAnimaliaBlattodeaBlattidae

Burmeister, 1838

CE14E705-046C-567C-9DA5-D4CCB55AAAE0

Periplaneta Burmeister, 1838: 502. Type species: Periplanetaamericana (Linnaeus, 1758). Shelford 1910: 17; Bey-Bienko 1950: 116; Princis 1966: 404; Asahina 1980: 103; Roth 1999: 168.
Cacerlaca Saussure, 1864: 71; Princis 1966: 405.
Paramethana Shelford, 1909: 309; Princis 1966: 473.
Shelfordella Adelung, 1910: 329; Princis 1966: 507; Bohn 1985: 39.

##### Diagnosis

**(based on species covered in this paper; *Periplanetas.s.*).** Sexual dimorphism indistinct or distinct. Pronotum subelliptical in male, subelliptical or campaniform in female. Tegmina and wings well developed in male, developed or reduced in female. Legs slightly slender. Abdomen with the first tergite unspecialized in male. Hind margin of supra-anal plate hyaline and concave in the middle; cerci long, apically tapering. Hind margin of subgenital plate slightly convex. ***Genitalia of male***: L1 weakly sclerotized with pubescence; hind margin of L4C nearly truncated; the caudal part of L2 with a long spine toward right; L3 with hlap weakly developed; the basal part of L4G constrict. R1H with two long spines at apex; the caudal part of R1G with a long and curved spine toward right. ***Genitalia of female***: Anterior arch (a.a.) with two symmetrical foot-shaped projections; spermathecal plate (sp.pl) nearly crescent-shaped; the enlarged part of spermatheca (sp.) curved, subelliptical or irregular; basivalvulae (bsv.) subelliptical; laterosternal shelf (ltst.sh.) with postero-lateral angle extended towards outer margin.

#### ﻿ Periplaneta arabica

Taxon classificationAnimaliaBlattodeaBlattidae

(Bey-Bienko, 1938)

A8B9A6F1-6F62-5BF0-875F-EBB4BAC21966

**Figure 2.**
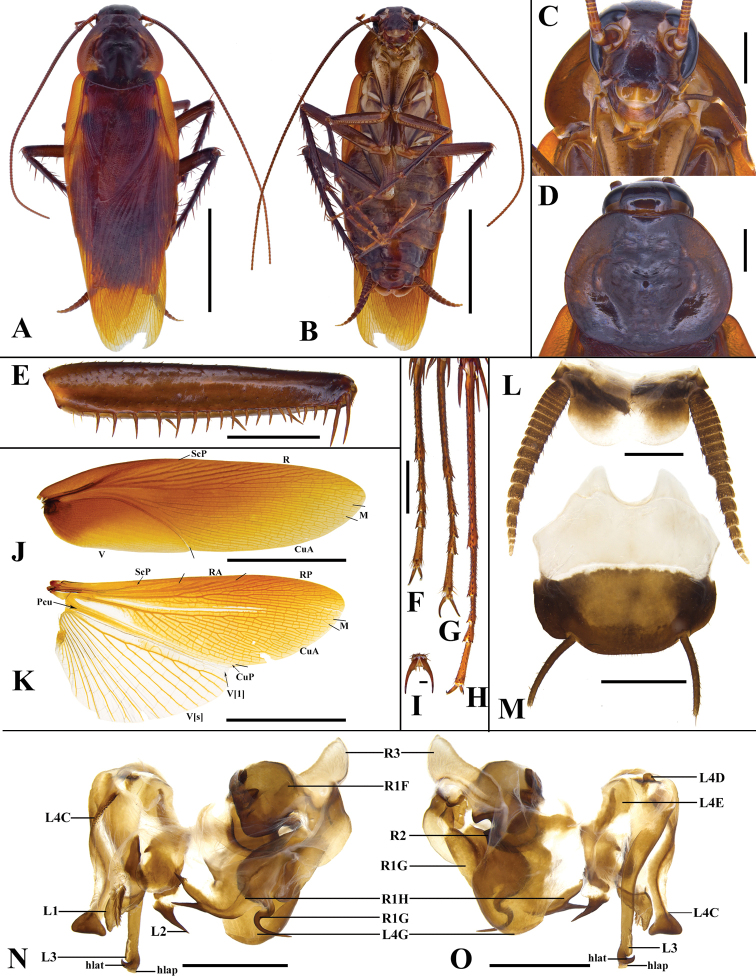
Male of *Periplanetaarabica* (Bey-Bienko, 1938) **A** habitus, dorsal view **B** habitus, ventral view **C** head **D** pronotum **E** front femur **F–H** tarsi (front, middle, hind) **I** arolium of hind leg **J** tegmen **K** hind wing **L** supra-anal plate **M** subgenital plate **N** phallomere, dorsal view **O** phallomere, ventral view. Scale bars: 10.0 mm (**A, B, J, K**); 2.0 mm (**C, D, E, F, G, H, L, M, N, O**); 0.5 mm (**I**).

**Figure 3.**
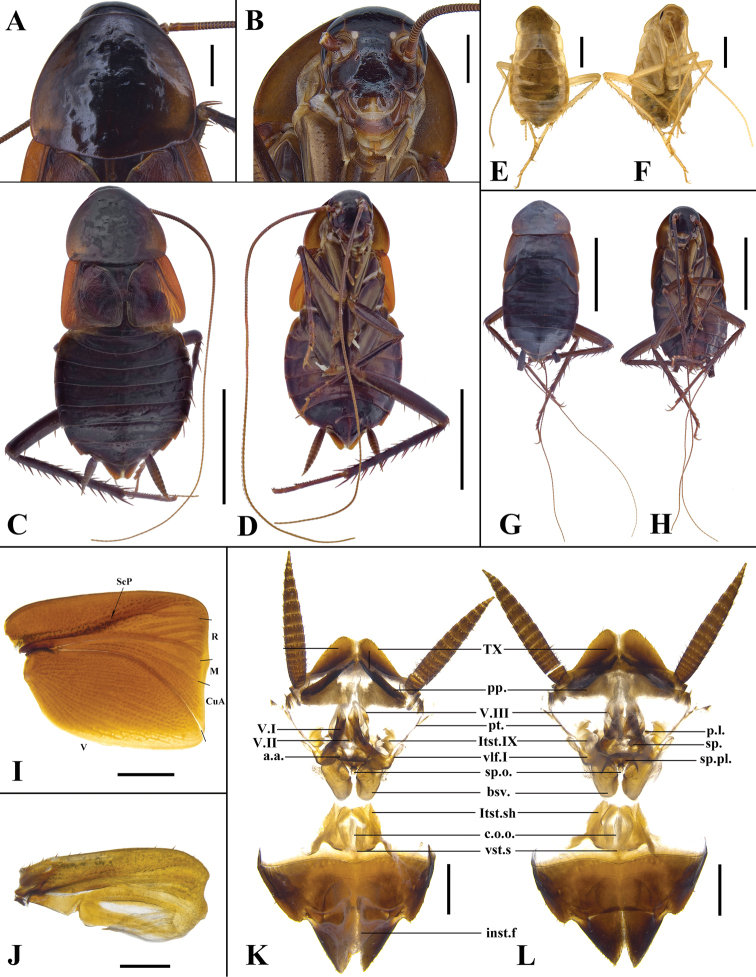
Female and nymph of *Periplanetaarabica* (Bey-Bienko, 1938) **A–D, I–L** female **A** pronotum **B** head **C** habitus, dorsal view **D** habitus, ventral view **I** tegmen **J** hind wing **K** genitalia, dorsal view **L** genitalia, ventral view **E–H** habitus of nymph, dorsal and ventral views. Scale bars: 10.0 mm (**C, D, G, H**); 2.0 mm (**A, B, E, F, I, K, L**); 1 mm (**J**).

**Figure 4.**
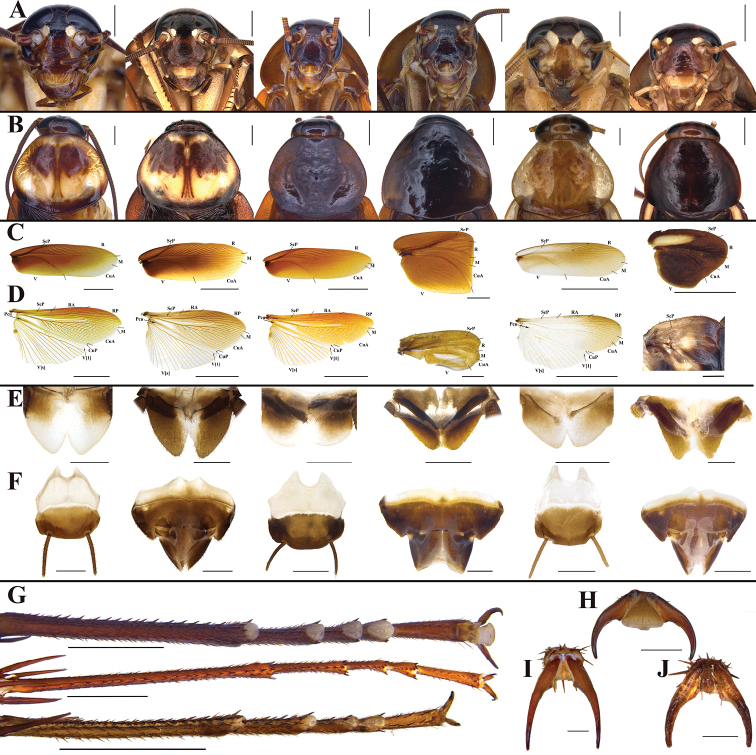
**A–F** In order from left to right, male of *P.americana*, female of *P.americana*, male of *P.arabica*, female of *P.arabica*, male of *P.lateralis*, female of *P.lateralis***A** heads **B** pronota **C** tegmina **D** hind wings **E** supra-anal plates **F** subgenital plates **G** hind tarsi (in order from top to bottom: *P.americana*, *P.arabica*, *P.lateralis*) **H–J** arolia of hind legs (in order: *P.americana*, *P.arabica*, *P.lateralis*). Scale bars: 10.0 mm (**C, D***P.americana*, males of *P.arabica* and *P.lateralis*); 2.0 mm (**A, B, E–G**, and females of *P.arabica* and *P.lateralis* in **C**); 1.0 mm (**D** females of *P.arabica* and *P.lateralis*); 0.5 mm (**H–J**).

Figs 2 , 3 , 4 (in part), 5 (in part), 6

Shelfordella arabica Bey-Bienko, 1938: 235 (Type locality: Mecca, Saudi Arabia); Bohn 2007: 87.
Blatta (Shelfordella) arabica : Princis 1966: 509.

##### Material examined

**(all deposited in SWU).** 6 males, 2 females and 7 nymphs; IRAN; Ilam Province: Dehloran county, near the border with Iraq, surroundings of Changuleh [33°0'49.37"N, 46°36'38.63"E, approximate coordinates], unnamed cave, II. 2020, A.H. Aghaei leg.

##### Diagnosis.

Combining the following characteristics, this species is easily distinguished from its congeners: 1) interocular space slightly wider than the interocellar space and less than interantennal space in male, interocular space wider than interantennal space in female; 2) tegmina of female reduced and nearly square; 3) legs slender, pulvilli and arolia absent; 4) hind margin not extending outward and slightly concave in the middle, forming an obtuse angle in supra-anal plate of male; 5) caudal part of L2 with a well sclerotized spine; 6) hlap weakly developed, but larger than that of the other two species; 7) distal part of R1H with two long spines and no serration.

##### Redescription.

***Measurements* (mm). Male.** Body length including tegmen: 30.6–36.4; body length: 24.2–27.3; pronotum length × width: 6.7–7.7 × 7.2–7.7; tegmen length × width: 24.9–29.2 × 4.6–5.4. **Female.** Body length: 23.5–25.5; pronotum length × width: 6.4–6.8 × 6.6–7.2; tegmen length × width: 4.4–6.4 × 6.6–7.3.

***Coloration*.** Body brown or reddish brown, eyes black, ocelli white; tegmina and wings yellowish brown.

**Male (Fig. 2). *Head* and *thorax*.** Vertex exposed. Interocular space slightly wider than the interocellar space, less than interantennal space. Antenna longer than the body (Fig. 2C). Pronotum subelliptical, with surface sparsely pubescent, the central part of anterior margin depressed, and hind margin slightly convex, the widest point approximately in the middle (Fig. 2D). Tegmina and wings well developed, exceeding the end of abdomen by about 5.3–7.7 mm. Tegmina with ScP strong, the distal part fusing with anterior branches of R; anterior branches of R with 2–4 bifurcations, posterior branches reaching the outer margin; the base of M distinct with 2–4 bifurcations; CuA slender with a few branches; V indistinct (Fig. 2J). Wings with ScP slender, the distal part of RA indistinct, RP slightly strong and distinct; M with 2–3 bifurcations at the end; CuA strong; V distinct (Fig. 2K). Legs (Fig. 2E–I) slender. Front femur type A_2_ (Fig. 2E). Hind metatarsus longer than the remaining segments combined (Fig. 2H). Pulvilli and arolia reduced; claws symmetrical (Fig. 2I). ***Abdomen*.** First tergite unspecialized. Supra-anal plate transversely broad, the lateral margins curved, and the hind margin slightly concave in the middle; the distal part less sclerotized and hyaline (Fig. 2L). Paraprocts (pp.) long strip-shaped and symmetrical. Cerci long, apically tapering. Subgenital plate nearly square, the hind margin slightly convex (Fig. 2M). Styli long, slender. ***Genitalia*** (Fig. 2N, O). L1 weakly sclerotized with pubescence. L4C with microspines on the lateral margin; the distal part expanded, hind margin nearly truncated. L2 curved and extended to left, the caudal part with a long spine toward right. L4D small (Fig. 2O). L4E flat. L3 unciform and well sclerotized; the base wide, downwardly tapering; the distal part bifurcated, hlap weakly developed. L4G elliptic with the basal part constricted. R1H flaky, with two long spines at the apex. The basal part of R1G broad, the distal with a long and curved narrow process toward right. R1F irregular and its outer margin thickened. R2 with a ridge-like projection in dorsal view. R3 located at the upper right, triangular and weakly sclerotized.

**Female (Fig. 3). *Head* and *thorax*.** Interocular space wider than interantennal space(Fig. 3B). Pronotum campaniform; anterior margin straight and hind margin convex, the widest point after the middle (Fig. 3A). Tegmina square, reduced and not reaching the first tergite of abdomen; lateral margins of tegmina truncated, forming nearly right angle with the anterior margin; R parallel to the anterior margin (Fig. 3I). Hind wings small lobe-like (Fig. 3J). ***Abdomen*** (Fig. 3K, L). Hind margin of tergum X (TX) with median invagination, and with a membranous line inside. Paraprocts (pp.) wide, nearly triangular. Subgenital plate divided; median with intersternal fold (inst.f.). ***Genitalia*** (Fig. 3K, L). First valve (v.I) sclerotized with dense punctures; the distal part hyaline, and the base fused with first valvifer (vlf.I). First valvifer short, parallel to paratergites (pt.) and laterosternite IX (ltst.IX). Paratergites slender and laterosternite IX irregular. Valvifer II (p.l.) annular. Second valve (v.II) small and flaky, the base fused, connecting with third valve (v.III) by membrane. Third valve (v.III) large and less sclerotized. Anterior arch (a.a.) wide and its central part with two symmetrical foot-shaped projections, surface with microtrichia. Spermathecal plate (sp.pl) well sclerotized and nearly crescent-shaped. Spermathecal opening (sp.o.) located at anterior margin of spermathecal plate. Spermatheca (sp.) divided into two branches, one branch with the distal part enlarged. Basivalvulae (bsv.) subelliptical with punctures. Postero-lateral angle of laterosternal shelf (ltst.sh.) extended towards outer margin. Vestibular sclerite (vst.s.) strip-shaped.

**Nymph.** Early instars are yellowish brown with ocelli and eyes small; in older nymphs, the body turns brown or reddish brown (Fig. 3E–H).

##### Distribution.

Saudi Arabia (Mecca); Yemen; United Arab Emirates; Oman; Iran (Ilam Province; new country record).

##### Remarks.

Bey-Bienko (1938) first documented and described this species based on a female specimen from Mecca, Saudi Arabia. Bohn (2007) provided some morphological characteristics of the male in the key to genera and species occurring in the United Arab Emirates. After checking the original description by Bey-Bienko (1938) and Bohn (2007) and the images of the type specimens, we consider *P.arabica* to be characterized by: 1) interocular space slightly wider than the interocellar space in female; 2) pronotum anterior margin straight and hind margin convex in female; 3) tegmina nearly square in female; 4) hind margin slightly concave in the middle to form an obtuse angle in supra-anal plate of male; these characteristics are present in our specimens as well. Therefore, we concluded that our material collected from western Iran should belong to *P.arabica*. Matching of individuals of different sexes and life stages was possible with DNA barcoding.

### ﻿Comparative morphology of *P.americana*, *P.arabica* and *P.lateralis*

A detailed morphological comparison of *P.americana*, *P.arabica* and *P.lateralis* was performed in this study. The following intraspecific variations were found in all three species: 1) the number of veins branches of wings; 2) the marks on disc of pronotum in male and female of *P.americana*; and 3) the color of the pronotum and abdominal tergite of female of *P.lateralis*.

### ﻿External morphological characters

The external morphological characteristics of *P.americana*, *P.arabica* and *P.lateralis* (Fig. 4) are compared in Table 2. Males of the three species have similar shapes of pronotum, wings, and supra-anal and subgenital plates, and lack tergite gland. Interocular space and interantennal space of females were both wider than the single eye spacing. The main differences among these three species are as follows: body size (i.e., *P.americana* > *P.arabica* > *P.lateralis*), tegmina and wings of females, and the presence or absence of arolia and pulvilli.

**Table 2.** Comparison of external morphological characters of males and females of three species of *Periplanetas.s.* Dimensions are in mm: mean±SEM (standard error of the mean). Abbreviations: Interocular space (IS); ocelli distance (OD); antennal sockets distance (ASD).

Species	*P.americana*		*P.arabica*		*P.lateralis*	
	male	female	male	female	male	female
Measured specimens (*N*)	23	15	6	2	17	13
Body length include tegmen (mm)	37.239±0.5960	33.327±0.3514	32.917±0.8388	–	24.206±0.2286	–
Body length	31.539±0.7966	30.113±0.6298	26.025±0.6537	24.500±1.0000	19.806±0.2397	20.715±0.3665
Distance comparison of IS, OD and ASD	IS ≤ OD < ASD	OD ≤ IS < ASD	OD < IS ≤ ASD	OD < ASD < IS	OD < IS < ASD	OD < IS ≤ ASD
Ocelli size	Medium	Medium	Medium	Small	Large	Medium
Pronotum shape	Subelliptical	Subelliptical	Subelliptical	Campaniform	Subelliptical	Campaniform
Tegmina	Well developed	Well developed	Well developed	Reduced and nearly square	Well developed	Reduced and nearly triangular
Hind wings	Well developed	Well developed	Well developed	Reduced and small lobed	Well developed	Reduced and fused to metanotum
Legs	Slightly slender	Slightly slender	Slender	Slender	Slightly slender	Slightly slender
Front femora	Type A2	Type A2	Type A2	Type A2	Type A2	Type A2
Pulvilli	Present	Present	Absent	Absent	Present	Present
Arolia	Medium	Medium	Absent	Absent	Minute	Minute
First tergite of abdomen	No tergite gland	–	No tergite gland	–	No tergite gland	–
Supra-anal plate’s shape	Hind margin extending outward and concave in the middle to forma sharp angle	Middle of hind margin deeply concave, forming one acute angle	Middle of hind margin concave and not extending	Hind margin not extending outward and slightly concave in the middle to form an obtuse angle	Hind margin extending outward and slightly concave in the middle to form an actue angle	Middle of hind margin forming an obtuse angle
Supra-anal plate’s sclerotization degree	The distal part less sclerotized and hyaline	Less sclerotized in the middle	The distal part less sclerotized and hyaline	Less sclerotized in the middle	The distal part less sclerotized and hyaline	Less sclerotized in the middle
Subgenital plate’s shape	Hind margin slightly convex	–	Hind margin slightly convex	–	Hind margin slightly convex	–

### ﻿Genitalia of male and female

**Figure 5.**
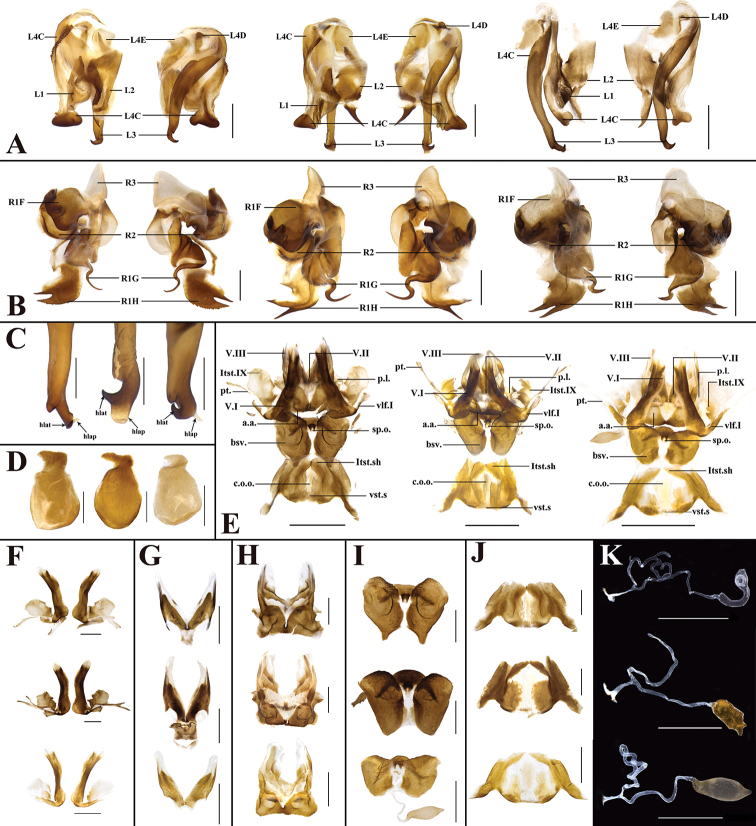
In order from left to right and top to bottom: *P.americana*, *P.arabica*, *P.lateralis***A** left phallomere, dorsal and ventral views **B** right phallomere, dorsal view **C**L3**D**L4G**E** overall female genitalia **F** first valve (v.I), first valvifer (vlf.I) and laterosternite IX (ltst.IX) **G** second valve (v.II) **H** third valve (v.III) and anterior arch (a.a.) **I** basivalvulae (bsv.) and spermathecal opening (sp.o.) **J** laterosternal shelf (ltst.sh.) **K** spermathecae (sp.). Scale bars: 2.0 mm (**E**); 1.0 mm (**A, B, D, F, G, H, I, J, K**); 0.5 mm (**C**).

As depicted in Fig. 5, the genitalia of *P.americana*, *P.arabica* and *P.lateralis* are highly similar in appearance but differ in the degree of development of the sclerites. In males (see *P.arabica* for detailed description), the results ranked in descending order are as follows: *P.lateralis* > *P.arabica* > *P.americana* for the pubescence density in L1, *P.arabica* > *P.americana* > *P.lateralis* for the sclerotization degree of spine in L2, and *P.arabica* > *P.lateralis* > *P.americana* for the development degree of the hlap in L3. In addition, there are certain differences in other aspects, for example, the basal margin of L4C in *P.americana* and *P.arabica* bears a row of microspines that is absent in *P.lateralis*, and a row of serration at the margin of R1H is present in *P.americana* but absent in *P.arabica* and *P.lateralis*. In females (see *P.arabica* for detailed description), the degree of development of some sclerites (i.e., valvifer II, laterosternite IX, basivalvulae and laterosternal shelf) is ranked as *P.americana* > *P.arabica* > *P.lateralis*. *Periplanetaamericana* differs from *P.lateralis* and *P.arabica* in the following characters: hind margin of basivalvulae (bsv.) with two symmetrical protrusions in the former, which is lacking in the latter two; furthermore, the enlargement of spermathecae (sp.) in *P.americana* is longer and curved (the degree of curvature varies among samples), but usually irregular in *P.arabica* and subelliptical in *P.lateralis*.

## ﻿Discussion

In recent years, molecular phylogenetic analyses have shown that *P.americana* has phylogenetic affinity with *P.lateralis* (Legendre et al. 2015; Bourguignon et al. 2018; Arab et al. 2020; Liao et al. 2021; Djernæs and Murienne 2022; Li et al. 2022; Deng et al. 2023), whereas *P. australasiae+P. fuliginosa+P. brunnea* would be the sister group to *Homalosilpha* (Liao et al. 2021; Djernæs and Murienne 2022; Deng et al. 2023). Deng et al. (2023) also included *P.japonica* and *P.karnyi*, neither of which clustered with *P.americana*. This inevitably raised doubts about the characteristics used in the past to distinguish *Periplaneta* and *Shelfordella*. Until recently, the development of tegmina and wings, pulvilli and arolia were usually considered the main diagnostic characters between these two genera (Adelung 1910; Bey-Bienko 1938; Bohn 1985). But, based on the phylogenetic results and some genital characteristics, Deng et al. (2023) considered *Shelfordella* as a synonym of *Periplaneta*. Considering the results of the current study, we also confirmed that *P.americana* differs significantly from *P.arabica* and *P.lateralis* in these characteristics. Our DNA-based analyses provided favorable evidence in the matching of females and males in all three species, as well as the pairing of adults and nymphs in *P.arabica*. Therefore, we had the possibility to compare males of these species and found that genitalia of both sexes of these three species were extremely similar, with differences in the developmental degree of sclerites. After a comparative morphological study on the genitalia of Blaberidae, Roth (1970, 1972, 1973) concluded that genital characters could be used as diagnostic characters for tribes, genera and groups. Until now, no such detailed genital comparison has been done in Blattidae, and our study might be helpful to solve the polyphyly of *Periplaneta* (Djernæs and Murienne 2022; Deng et al. 2023). In addition, considering the close relationship of *P.americana* and *P.lateralis* and the fact that both *P.arabica* and *P.lateralis* originated from West Asia (Beccaloni 2014), we speculate that *P.americana* might have originated in this region as well, and later dispersed naturally or was introduced by humans to other parts of the world, before gradually becoming a notorious indoor pest.

**Figure 6.**
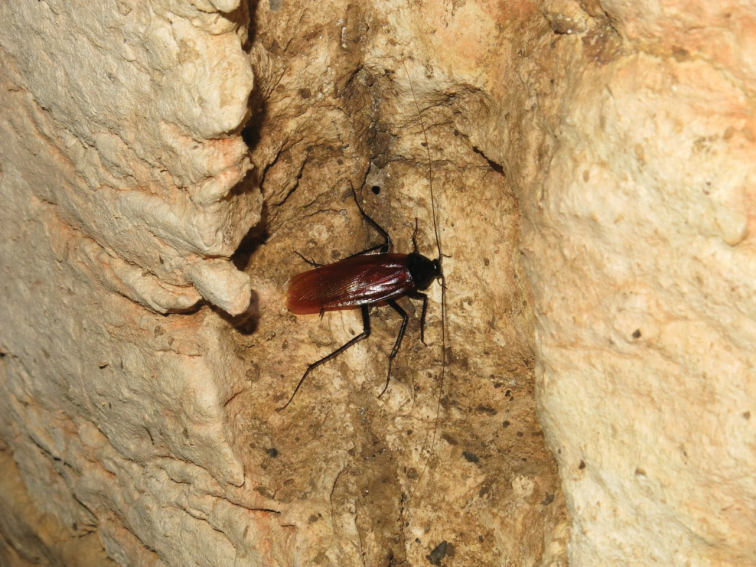
Male of *P.arabica* from a cave in Ilam, Iran. Photographed by Alireza Zamani.

Before the extensive usage of molecular data in cockroach systematics, most genera of Blattinae were established mainly on the basis of external morphological characters. As a matter of fact, the wings, pulvilli and arolia of cockroaches are heavily influenced by the environment and lifestyle (Arnold 1974; Bell et al. 2007). In deserts, a cave-dwelling lifestyle is a survival strategy for cockroaches (Roth and Willis 1960). Material of *P.arabica* reported in this study were sampled from a natural cave in western Iran (Fig. 6), which has a subtropical desert climate (Burstyn et al. 2019). Morphologically, slender antennae and legs, absent pulvilli and arolia, lighter body and very small ocelli of early instars are consistent with the convergent evolution of cave-dwelling species (Bell et al. 2007; Lucañas and Lit 2016). In contrast, *P.americana* has well-developed tegmina and wings, and developed pulvilli and arolia in both sexes, which could be favorable to facilitate its dispersal and climbing ability (Clemente and Federle 2008), and also beneficial for this species to colonize other environments (e.g., human settlements, tree trunks in the wild, landfills, and shallow caves with abundant guano; Lucañas et al. 2022) in search for food. Therefore, influenced by their environment and lifestyle, these three species have maintained a high similarity in genitalia, but greatly diverged in external morphology.

## Supplementary Material

XML Treatment for Periplaneta

XML Treatment for Periplaneta arabica

## Supplementary materials

Supplementary material 1 Genetic divergence of distances calculated by K2P model method using cytochrome c oxidase subunit I (COI) gene sequences in MEGA Data type table (excel file) https://binary.pensoft.net/file/807594This dataset is made available under the Open Database License (http://opendatacommons.org/licenses/odbl/1.0/). The Open Database License (ODbL) is a license agreement intended to allow users to freely share, modify, and use this Dataset while maintaining this same freedom for others, provided that the original source and author(s) are credited.Xin-Xing Luo, Qian-Qian Li, Alireza Zamani, Yan-Li Che, Zong-Qing Wang

Supplementary material 2 Interspecific genetic divergence of distances calculated by K2P model method using cytochrome c oxidase subunit I (COI) gene sequences in MEGA Data type table (excel file) https://binary.pensoft.net/file/807595This dataset is made available under the Open Database License (http://opendatacommons.org/licenses/odbl/1.0/). The Open Database License (ODbL) is a license agreement intended to allow users to freely share, modify, and use this Dataset while maintaining this same freedom for others, provided that the original source and author(s) are credited.Xin-Xing Luo, Qian-Qian Li, Alireza Zamani, Yan-Li Che, Zong-Qing Wang
